# Systematic review and meta-analysis of preventive interventions for eating disorders

**DOI:** 10.1186/2050-2974-3-S1-O35

**Published:** 2015-11-23

**Authors:** Long KD Le, Jan Barendregt, Phillipa Hay, Cathrine Mihalopoulos

**Affiliations:** 1 Deakin Health Economics, Population Health Strategic Research Centre, Deakin University, Australia; 2 Epigear International Pty Ltd, Australia; 3 School of Public Health, The University of Queensland, Australia; 4 School of Medicine and Centre for Health Research, University of Western Sydney, Australia; 5 School of Medicine, James Cook University, Australia

## Background

Eating disorders (EDs) are serious mental disorders which have significant physical and psychological impacts. The aim of this study is to determine whether ED prevention programs are effective across the age spectrum.

## Method

Electronic databases (including the Cochrane Controlled Trial Register, MEDLINE, PsychInfo, EMBASE, and Scopus) were searched for published randomised controlled trials (RCTs) designed for preventing ED. Included trials were classified as both broader prevention strategies (eg. universal) and more specific prevention program (eg. cognitive behavioural therapy (CBT)). Quantitative meta-analyses were conducted.

## Results

A total of 3790 titles were retrieved via the initial search strategy. Ninety-eight RCTs were included in the meta-analysis. Most of studies used self-report measurements of ED risk factors, and did not report final outcomes of cases of ED prevented. For universal prevention, media literacy (ML) interventions demonstrated a significant reduction in ED risk factors (e.g. weight and shape concern) at up to 30 months after interventions for both male and female adolescents. For selective prevention CBT (delivered by class or online) and ML interventions have significantly reduced bulimic symptoms at 3- to 8- month follow up (SMD -0.31, 95% CI: -0.58 to -0.03), and thin-ideal internalisation at 3-month follow up (SMD -1.79, 95% CI: -3.14 to -0.45) , respectively. CBT and cognitive dissonance (CD) interventions for indicated prevention showed strong evidence and substantial effect in reducing both ED risk factor and ED symptoms. A healthy weight program which was designed to prevent both ED and obesity as an indicated prevention intervention was found to significantly reduce ED risk factors and body mass.

## Conclusions

The study supported the conclusions that indicated prevention interventions using CBT and CD approaches are effective for ED prevention while universal and selective prevention using media literacy and selective prevention using CBT are promising. Combined ED and obesity prevention interventions require further research.

